# A mechanism linking perinatal 2,3,7,8 tetrachlorodibenzo-*p*-dioxin exposure to lower urinary tract dysfunction in adulthood

**DOI:** 10.1242/dmm.049068

**Published:** 2021-07-27

**Authors:** Anne E. Turco, Steven R. Oakes, Kimberly P. Keil Stietz, Cheryl L. Dunham, Diya B. Joseph, Thrishna S. Chathurvedula, Nicholas M. Girardi, Andrew J. Schneider, Joseph Gawdzik, Celeste M. Sheftel, Peiqing Wang, Zunyi Wang, Dale E. Bjorling, William A. Ricke, Weiping Tang, Laura L. Hernandez, Janet R. Keast, Adrian D. Bonev, Matthew D. Grimes, Douglas W. Strand, Nathan R. Tykocki, Robyn L. Tanguay, Richard E. Peterson, Chad M. Vezina

**Affiliations:** 1 Molecular and Environmental Toxicology Center, University of Wisconsin-Madison, Madison, WI 53705, USA; 2 Department of Comparative Biosciences, University of Wisconsin-Madison, Madison, WI 53705, USA; 3 Department of Environmental and Molecular Toxicology, Oregon State University, Corvallis, OR 97331, USA; 4 Department of Urology, University of Texas Southwestern, Dallas, TX 75390, USA; 5 School of Pharmacy, University of Wisconsin-Madison, Madison, WI 53705, USA; 6 Cellular and Molecular Pharmacology, University of Wisconsin-Madison, Madison, WI 53705, USA; 7 Department of Urology, University of Wisconsin-Madison, Madison, WI 53705, USA; 8 Department of Animal and Dairy Sciences, University of Wisconsin-Madison, Madison, WI 53705, USA; 9 Department of Anatomy and Physiology, University of Melbourne, Melbourne, VIC 3010, Australia; 10 Department of Pharmacology, University of Vermont, Burlington, VT 05405, USA; 11 Department of Pharmacology and Toxicology, Michigan State University, East Lansing, MI 58823, USA

**Keywords:** Dioxin, Noradrenergic, Prostate, Smooth muscle, Artemin, Lower urinary tract dysfunction

## Abstract

Benign prostatic hyperplasia/lower urinary tract dysfunction (LUTD) affects nearly all men. Symptoms typically present in the fifth or sixth decade and progressively worsen over the remainder of life. Here, we identify a surprising origin of this disease that traces back to the intrauterine environment of the developing male, challenging paradigms about when this disease process begins. We delivered a single dose of a widespread environmental contaminant present in the serum of most Americans [2,3,7,8 tetrachlorodibenzo-*p*-dioxin (TCDD), 1 µg/kg], and representative of a broader class of environmental contaminants, to pregnant mice and observed an increase in the abundance of a neurotrophic factor, artemin, in the developing mouse prostate. Artemin is required for noradrenergic axon recruitment across multiple tissues, and TCDD rapidly increases prostatic noradrenergic axon density in the male fetus. The hyperinnervation persists into adulthood, when it is coupled to autonomic hyperactivity of prostatic smooth muscle and abnormal urinary function, including increased urinary frequency. We offer new evidence that prostate neuroanatomical development is malleable and that intrauterine chemical exposures can permanently reprogram prostate neuromuscular function to cause male LUTD in adulthood.

## Abstract (precis)

**Summary:** We describe a new mechanism of benign prostate disease, initiated by fetal chemical exposure, which durably increases prostatic noradrenergic axon density and causes smooth muscle hyperactivity and urinary voiding dysfunction.

## INTRODUCTION

Benign prostatic hyperplasia/lower urinary tract dysfunction (LUTD) is nearly universal in aging men. If untreated, LUTD can cause permanent bladder dysfunction, renal injury and even death (Lepor, 2005). LUTD is linked to excessive prostate/bladder neck smooth muscle tone, which causes outlet resistance and is the target of α-adrenergic receptor blockers, a first-line therapy for male LUTD. Aging-related processes such as benign prostatic enlargement have been the sole focus of male LUTD research for decades. Almost no attention has been directed to the first three decades of postnatal life or the intrauterine environment, where chemical exposures and other factors are known risk modifiers for a growing spectrum of aging-related diseases, including hypertension, diabetes, cancer and others (Potischman and Troisi, 1999; Langley-Evans, 2001; Simmons et al., 2001).

2,3,7,8 tetrachlorodibenzo-*p*-dioxin (TCDD) is a widespread contaminant (Gupta et al., 2006). It is the most potent agonist of the aryl hydrocarbon receptor (AHR), a ligand-induced transcription factor that mediates most of TCDD's biological actions (Okey et al., 1994). TCDD is used experimentally to model AHR activation by other common environmental contaminants, including polychlorinated dibenzofurans (PCDFs), polychlorinated dibenzodioxins (PCDDs), polychlorinated biphenyls (PCBs), polycyclic aromatic hydrocarbons (PAHs) and heteroaromatic amines (HAAs) (Powell and Ghotbaddini, 2014; Organtini et al., 2017). These chemicals are introduced into the environment through manufacturing and combustion processes, and human exposure occurs through dietary consumption of contaminated foods, such as red meat, dairy and fish, and other sources, such as air pollution (Powell and Ghotbaddini, 2014). Dioxin-like AHR agonists are ubiquitous in the serum of pregnant women, where they can pass through the placenta to expose the developing fetus (Wang et al., 2009; Woodruff et al., 2011). The average background body burden of TCDD equivalents in the general human population is 0.013 µg/kg (DeVito et al., 1995). Human populations with documented exposure have higher body burdens of TCDD equivalents, 0.096-7.0 µg/kg, and adverse health effects have been associated with them (DeVito et al., 1995). A body burden of TCDD equivalents of 2.13 µg/kg in pregnant women is associated with decreased birth weight (Sunahara et al., 1987; Lucier, 1991), decreased growth (Guo et al., 1994) and delayed developmental milestones (Rogan et al., 1988; Chen et al., 1992) in their offspring. The TCDD dose used in pregnant mice in the present study, 1.0 µg/kg, is in the same maternal dose range of TCDD equivalents that disrupts child health.

We offer new evidence that the LUTD disease process can be initiated far earlier than previously considered. Using bulk RNA sequencing (RNA-seq) of the fetal prostate, we discovered that TCDD exposure coinciding with the onset of prostate innervation (Turco et al., 2019) increases abundance of the neurotrophic/survival factor artemin (*Artn*), which was previously shown to be critical for noradrenergic axon development (Baloh et al., 1998; Honma et al., 2002). TCDD rapidly increases noradrenergic axon density in the fetal prostate. Whereas fetal changes in innervation are often resolved by axon pruning, TCDD-mediated prostate hyperinnervation is durable and continues into adulthood, when it is functionally coupled to prostate smooth muscle hyperactivity and LUTD. Our results draw new attention to the perinatal environment as a factor that shapes adult male voiding function and serves as a risk modifier for LUTD in advancing age.

## RESULTS

### Fetal TCDD exposure changes urinary voiding function in adulthood

**Fig. 1.**
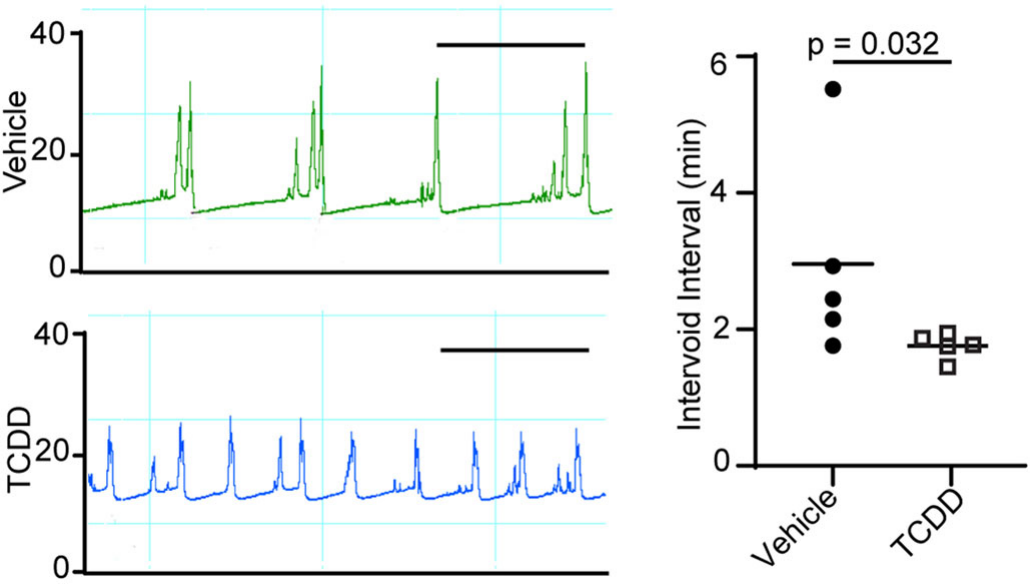
**A single dose of TCDD, delivered to male mice during development, changes urodynamic voiding behavior in adulthood.** C57BL/6J mouse fetuses were exposed to a single dose of TCDD [1 µg/kg maternal dose, orally (po)] or vehicle (5 ml/kg corn oil, po, control) on E13 and were evaluated by cystometry between P90 and P98. Mice were anesthetized, a cystostomy catheter was passed through the bladder dome, and saline was infused at a rate of 1.5 ml/h while continuously measuring bladder pressure in response to filling and emptying. The left panel shows representative pressure versus time traces (top, vehicle; bottom, TCDD), with each peak indicating bladder contraction during a voiding event. The *y*-axes indicate changes in intravesical pressure (mmHg). Three to five consecutive voids were used to quantify mean responses. *In utero* and lactational TCDD exposure decreases the intervoid interval. Results are from five mice per group, representing at least three independent litters. Scale bars: 2.5 min. The intervoid intervals are quantified in the right panel. Unpaired Student's *t*-test was used to identify differences between groups after a log transformation to normalize distribution. Results are from five mice per group, representing at least three independent litters.

The intrauterine environment has been linked to multiple aging-related diseases, and maternal chemical exposures during pregnancy are risk modifiers for these diseases. For example, AHR activation by TCDD or related chemicals in the fetus has been linked to increased risk for eczema (Ye et al., 2018), autoimmune diseases (Gogal and Holladay, 2008), neurodevelopmental disorders (Pham et al., 2019) and impairment of mammary gland differentiation (Vorderstrasse et al., 2004), as well as others. To test whether fetal TCDD exposure changes urinary function in adult male mice, a single dose of TCDD (1 µg/kg, oral maternal dose) was given at embryonic day (E)13.5 (resulting in continuous exposure beginning *in utero* and throughout lactation), and urinary function was evaluated by cystometry in anesthetized male mice on postnatal day (P)90-P98. TCDD exposure resulted in significantly reduced intervoid interval (Fig. 1), whereas peak voiding pressure was not significantly changed (Fig. S1A).

### Fetal TCDD exposure increases the sensitivity of adult prostatic smooth muscle to electrically evoked contraction

**Fig. 2.**
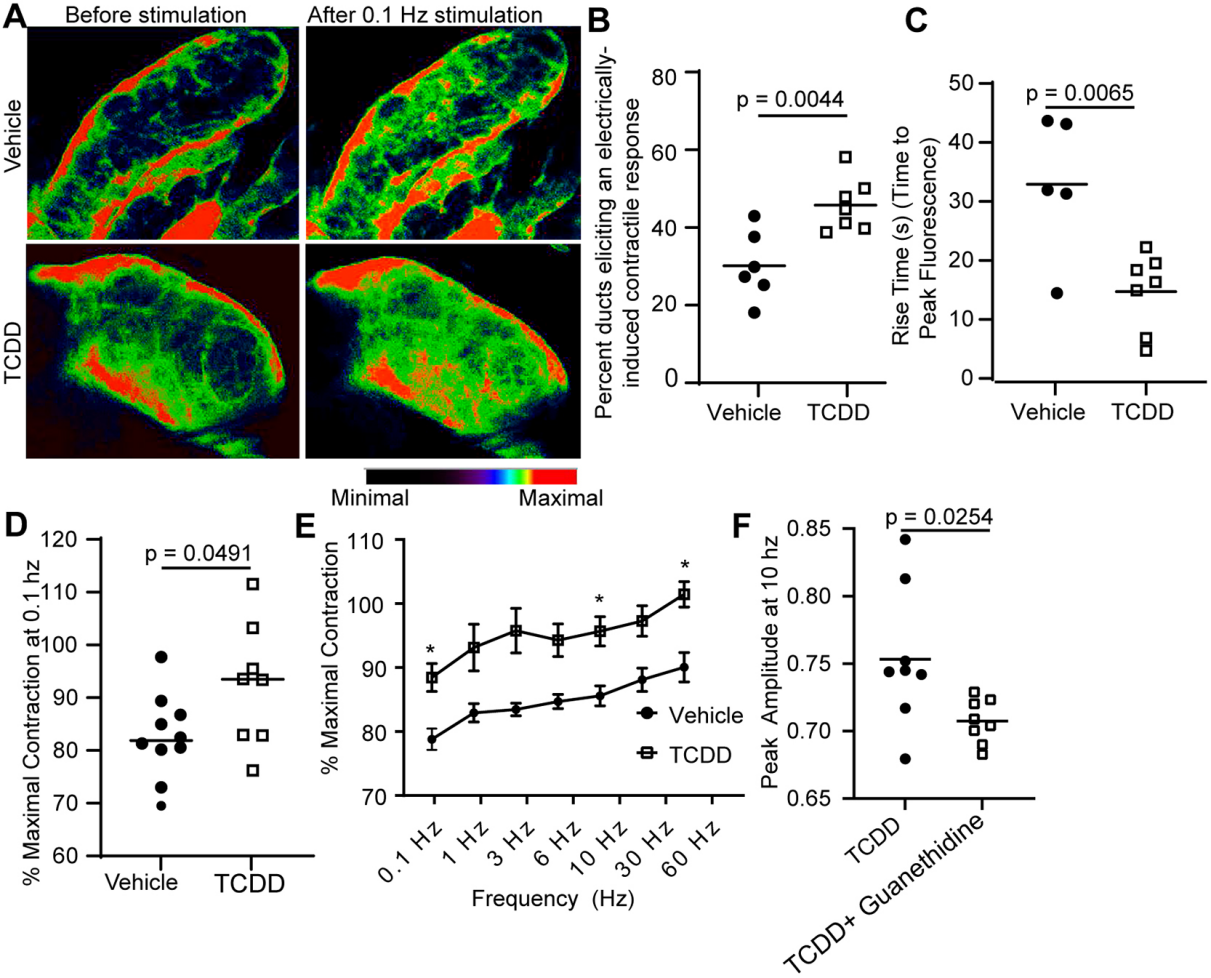
**A single dose of TCDD, delivered to male mice during the perinatal period, increases adult prostatic smooth muscle responsiveness to field stimulation.** E13 *Myh11^cre^/+*;*GCaMP5g/+* male mice were exposed to TCDD (1 µg/kg maternal dose, po) or vehicle (5 ml/kg, po, control). For A-D, prostates were collected between P90 and P98 and placed in a perfusion chamber with 37°C HEPES buffer. (A) Representative fluorescent images were captured at baseline and (contracted) 40 s after a 0.1 Hz stimulus*.* (B,C) *In utero* and lactational TCDD exposure of *Myh11*^cre^/+;GCaMP5g/+ mice increases the percentage of prostatic ducts that elicit a change in diameter (contraction) in response to a 0.1 Hz stimulus (B) and reduces the time needed to reach peak fluorescence (C). (D-F) In separate experiments, field stimulation was applied to tensioned adult dorsal prostate tissue incubated in 37°C Krebs buffer. (D) *In utero* and lactational TCDD exposure increases the maximum response to 0.1 Hz stimulation compared to vehicle-exposed dorsal prostate tissue. (E) *In utero* and lactational TCDD exposure increases the contractile response to 0.1, 10 and 60 Hz stimuli compared to control with a repeated measures two-way ANOVA (*P≤0.05). (F) Pretreatment with 10 µM guanethidine decreased the maximal response of TCDD-exposed tissues to field stimulation. The peak amplitude at 10 Hz is shown as an example indicating that axons are responsible for TCDD-induced sensitization. Results are from six to ten mice per group, representing at least three independent litters. Unpaired Student's *t*-test was used to identify differences between groups.

TCDD exposure is associated with prostatic smooth muscle hypertrophy in adult mice and rhesus macaques (Arima et al., 2010; Ricke et al., 2016; Turco et al., 2020). Smooth muscle hyperactivity can drive hypertrophy; therefore, we tested whether TCDD exposure increases nerve-evoked prostate muscle contraction. We introduced a genetically encoded calcium sensor (GCamP) into prostate smooth muscle to quantify calcium transients. *Myh11^cre^/+*;*GCaMP5g/+* male mouse fetuses were exposed to TCDD or vehicle (control) on E13.5, as previously described, and euthanized at P90-P98. Excised lower urinary tracts (anterior lobes removed and dorsal prostate lobes attached to urethra) were transferred to a heated perfusion bath, and prostate preparations were stimulated with trains of 0.5 ms pulses at 60 V with 0.1 Hz (below the frequency leading to half-maximum response) (Lau et al., 1998) while continuously recording tissue fluorescence (Movies 1 and 2). Stimulated tissues increase in fluorescence and eventually return to baseline fluorescence*.* TCDD significantly decreases the rise time in field stimulation-induced calcium fluorescence, with TCDD-treated tissues reaching peak fluorescence (maximal contraction) more quickly than controls. Rise time is an index of muscle sensitivity; peak fluorescence does not change with treatment but the time to reach the maximal contraction is significantly less in TCDD-exposed animals. *In utero* and lactational TCDD exposure also increased the percentage of prostate ducts with detectable changes in ductal diameter in response to field stimulation (Fig. 2).

We complemented imaging experiments with isometric contractility assays to measure prostate smooth muscle tension in response to graded field stimuli. TCDD exposure significantly increased dorsal prostate tension in response to 0.1, 3, 10 and 60 Hz stimuli (Fig. 2E). We tested whether noradrenaline specifically mediates the TCDD-induced response in the dorsal prostate by blocking the release of catecholamines (including norepinephrine) from autonomic axons with the addition of sympatholytic guanethidine to the tissue bath (1.983 µg/ml) (Lau et al., 1998). Guanethidine pretreatment significantly attenuated TCDD-mediated enhancement of prostate field stimulation (Fig. 2F). The residual response is potentially due to a cholinergic-mediated contraction (White et al., 2010). The actions of TCDD also exhibited regional specificity for prostate and prostatic urethra, which are under noradrenergic control. The contractile response of the bladder, which is primarily mediated by cholinergic rather than noradrenergic mechanisms, was unchanged by TCDD exposure (Fig. S1B).

From these results, we surmised that TCDD could act through two mechanisms: it could directly enhance noradrenergic transmission, or it could increase the sensitivity of prostate smooth muscle adrenoreceptors to agonists. Dorsal prostate tension response to increasing concentrations of phenylephrine (0.0001-200 µM) is unchanged after TCDD exposure, ruling out a change in smooth muscle adrenergic sensitivity (Fig. S1C). Thus, we conclude that TCDD exposure enhances the magnitude and duration of adrenergic outflow within the prostate.

### A single dose of TCDD at E13.5 increases prostate noradrenergic axon density beginning in the fetal period and persisting into adulthood

**Fig. 3.**
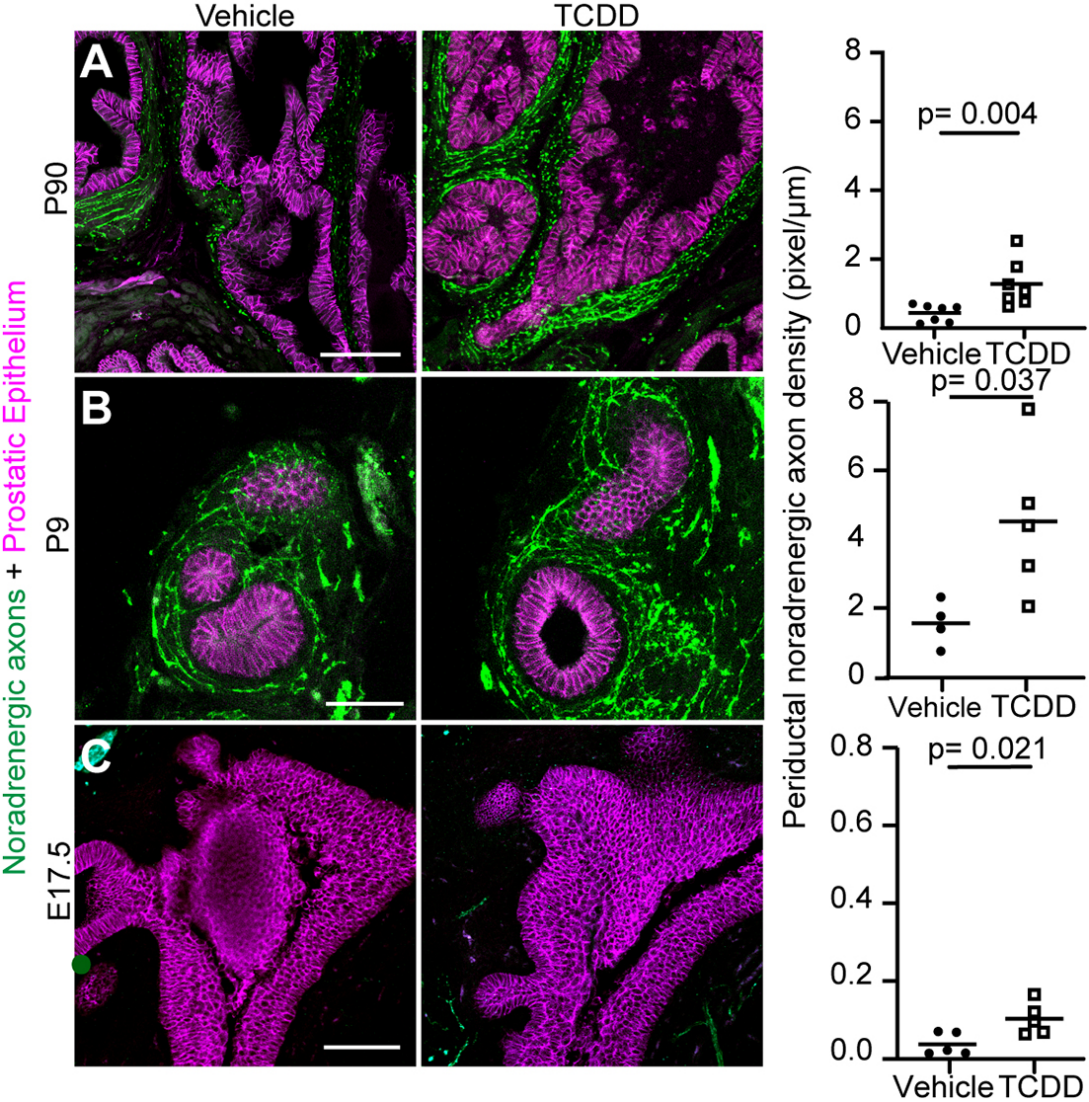
**A single perinatal dose of TCDD increases noradrenergic axon density in the prostatic periductal region beginning in the fetal period and persisting into adulthood.** (A-C) Male mice were exposed to TCDD (1 µg/kg, po) or vehicle (5 ml/kg, po, control) at E13.5, and noradrenergic axon density was assessed immunohistochemically in a dorsal prostate tissue sections (three non-serial sections per animal) at P90 (seven mice per group) (A), P9 (four to five mice per group) (B) and E17.5 (five mice per group) (C). Results are from at least three independent litters per group. An antibody against tyrosine hydroxylase (TH; green) was used to identify noradrenergic axons and an antibody against cadherin 1 (CDH1; magenta) was used to localize prostatic epithelium. TH^+^ axons were quantified in the area extending 10 µm from prostatic epithelium. Vehicle groups at P9-P90 differ in density due to different tissue structures in neonatal versus adult prostate. Scale bars: 50 µm for P50 and P9, 100 µm for E17.5. Unpaired Student's *t*-test was used to identify differences between groups, and *P*≤0.05 was considered significant. Results are from four to seven mice per group, representing at least three independent litters.

Men with LUTD often present with autonomic imbalance characterized by decreased high-frequency heart rate variability; the mechanism for this imbalance is not known but could derive from an underlying difference in neuroanatomy (Choi et al., 2010). Recent studies demonstrate that prostate axon density is modifiable. For example, the prostate tumor microenvironment increases the density of parasympathetic cholinergic axons (Magnon et al., 2013). We next tested whether TCDD exposure influences prostate noradrenergic axon density. Mice were treated with TCDD or vehicle, as previously described, and adult (P90) prostate tissue sections were evaluated by immunostaining. Noradrenergic axons were labeled with antibodies against tyrosine hydroxylase (TH) and quantified in the 10 µm periductal region of prostate stromal tissue extending outward from cadherin 1 (CDH1)-stained prostate epithelium (Fig. 3A). We focused specifically on the periductal region, as it contains most of the prostate periductal smooth muscle (Turco et al., 2019). *In utero* and lactational TCDD significantly increased noradrenergic axon density in the dorsal prostate, suggesting that TCDD modifies axon patterning during organ development (Fig. 3A).

Noradrenergic axons are first evident in the mouse prostate at E13.5 (Turco et al., 2019), coinciding with the initiation of TCDD exposure. We next tested whether TCDD acts through a developmental mechanism to increase prostate noradrenergic axon density. To pinpoint the stage of life when TCDD first increases noradrenergic axon density, mice were treated at E13.5 with TCDD or vehicle, and axons were quantified at two time points: (1) P9, coinciding with active prostate ductal branching morphogenesis and when noradrenergic axons are most dense (Turco et al., 2019); and (2) E17.5, during prostate bud initiation (Lin et al., 2004). TCDD exposure significantly increased noradrenergic axon density at both P9 (Fig. 3B) and E17.5 (Fig. 3C). Thus, TCDD exposure beginning on E13.5 increases prostate noradrenergic axon density during the fetal and perinatal periods, and this change persists at least to adulthood.

TCDD actions on prostate axon density are not caused by changes in prostate size, as TCDD did not significantly change ventral, anterior, or dorsal prostate weights or body weights (Fig. S1D,E). Furthermore, TCDD actions on prostate innervation appear to be selective for noradrenergic axons. *In utero* and lactational TCDD exposure does not change immunopositive axon pixel densities at P90, P9 or E17.5 for markers of sensory axons [calcitonin gene-related peptide (CGRP)], a pan-axonal marker [βIII tubulin (TUBB3)] or cholinergic axons [vesicular acetylcholine transporter (VAChT; also known as SLC18A3)] (Figs S2-S4).

### Fetal TCDD exposure increases prostate mRNA abundance of *Artn*, a neurotrophic factor linked to axon recruitment

**Fig. 4.**
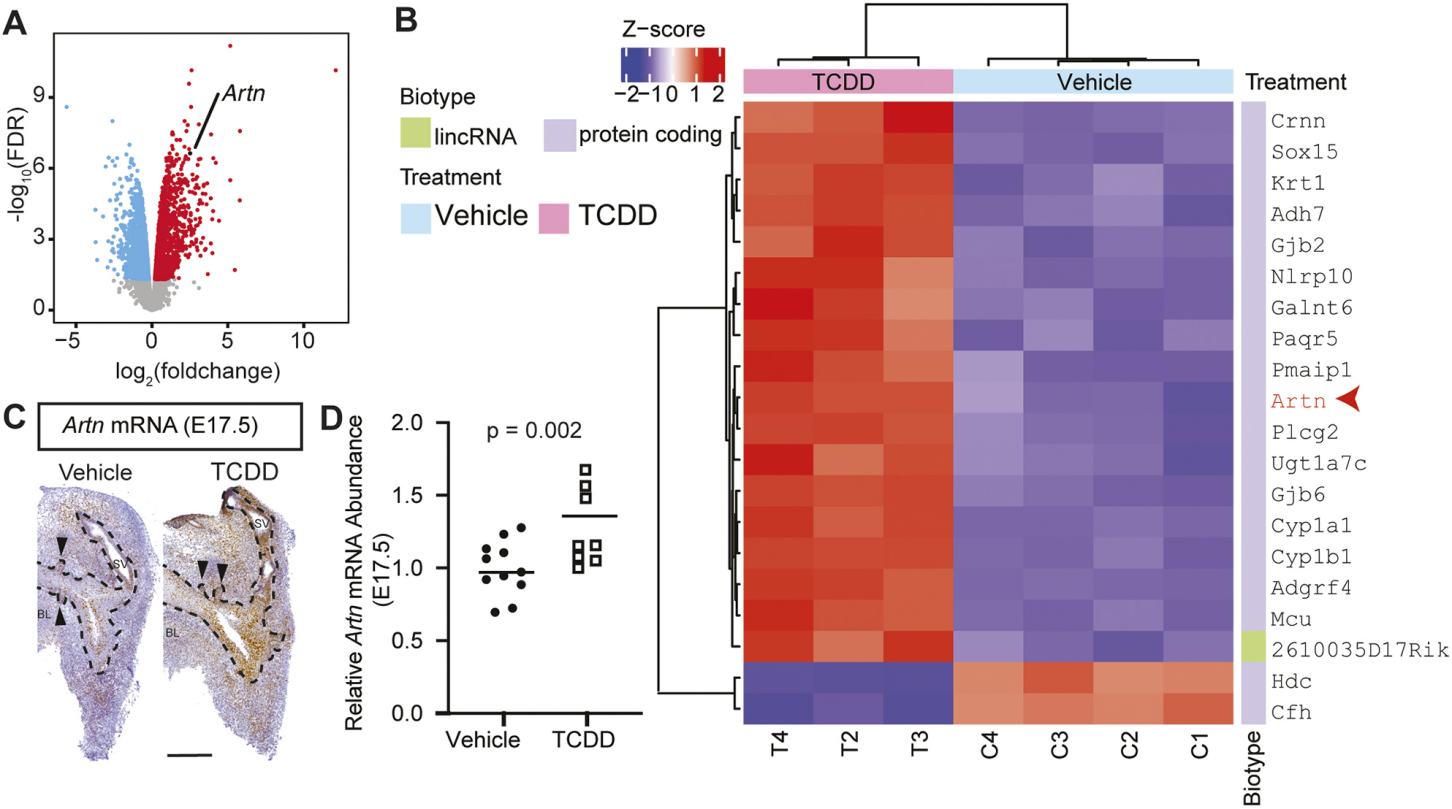
***In utero* TCDD exposure, coinciding with the beginning of prostatic neuroanatomical development, increases mRNA abundance of the GDNF member *Artn* in the fetal prostate.** Male mice were exposed to TCDD (5 µg/kg, po maternal dose) or vehicle (5 ml/kg, po maternal dose, control) on E13.5, and urogenital sinus (UGS) epithelium was collected for RNA-seq on E16.75. (A) Volcano plot showing significantly up- and downregulated genes from TCDD exposure. Red indicates upregulated genes, blue indicates downregulated genes, and *Artn* is identified in black (*n*=3-4, FDR-adjusted *P*≤0.05). (B) The top 20 differentially expressed genes, ordered by FDR-adjusted *P*-value, included *Artn*. (C) *In situ* hybridization localized *Artn* mRNA (brown staining) to UGS epithelium and periprostatic bud mesenchyme of E17.5 (control) C57BL/6J male fetuses. BL, bladder; SV, seminal vesicle; arrowhead, prostatic bud; the dashed line indicates the boundary between UGS epithelium and mesenchyme. (D) Real-time RT-PCR indicated that a single maternal dose of TCDD (1 µg/kg, po) on E13.5 significantly increased *Artn* mRNA abundance in the E17.5 male UGS compared to vehicle. Scale bar: 250 µm. Unpaired Student's *t*-test was used to assess differences in RT-PCR data, and differential expression of genes was determined using functions from edgeR, the Cox–Reid profile-adjusted likelihood method to calculate dispersions, empirical Bayes quasi-likelihood *F*-tests, and a version of the *t*-tests relative to a threshold (TREAT) method. Results are from eight to 11 male fetuses per group, representing three independent litters, and *P*≤0.05 was considered significant.

Because TCDD exposure increases prostate axon density during the period when axons project to the prostate, we used RNA-seq to survey the genomic response to TCDD and test whether TCDD induces neurotrophins/neurotrophic factors. We focused on the epithelium, because it is a considerable source of neurotrophins/neurotrophic factors in other developing organs (de Groat, 2013) and shifted the timing of TCDD exposure to coincide with prostate specification, the period when axons are actively recruited (Vezina et al., 2008a,b). We also increased the TCDD dose to increase the dynamic range of the transcriptional response. A single maternal dose of 5 µg/kg TCDD was delivered on E15.5, and fetal prostate epithelium was collected at E16.75 for RNA-seq analysis (Fig. 4A,B). TCDD exposure initially led to the differential expression of 3276 genes in fetal prostate epithelium (Fig. 4A; Dataset 1). To focus on the most biologically relevant changes while reducing the number of false positives, differential expression was determined using a threshold log_2_(1.5) fold-change cutoff, which resulted in 573 significantly differentially expressed genes [*n*=3-4, Benjamini–Hochberg false discovery rate (FDR)-adjusted *P*<0.05; Dataset 1]. *Artn*, a member of the glial cell-derived neurotrophic factor family that recruits noradrenergic axons, was among the top 20 most differentially expressed genes by TCDD ordered by FDR-adjusted *P*–value [log_2_(fold change)=2.51, *n*=3–4, FDR-adjusted *P*=1.55×10^−6^; Fig. 4B; Dataset 1]. To validate the RNA-seq results and enhance rigor, we copied the TCDD dose and timing used in Figs S1-S3 (1 µg/kg maternal dose at E13.5) to evaluate TCDD-mediated changes in *Artn*. We used RNAScope to localize *Artn* mRNA in E17.5 urogenital sinus (UGS) epithelium of TCDD and vehicle samples and peri-epithelial mesenchyme (Fig. 4C), the same regions in which fetal TCDD exposure activates the AHR (Vezina et al., 2008a,b). We used reverse transcriptase polymerase chain reaction (RT-PCR) to confirm that TCDD increases *Artn* mRNA abundance in the E17.5 male UGS (Fig. 4D). We also showed that the TCDD-mediated increase in *Artn* mRNA abundance is transient; it is limited to the fetal period and is no longer differentially regulated in the P9 prostate (Fig. S5).

**Fig. 5.**
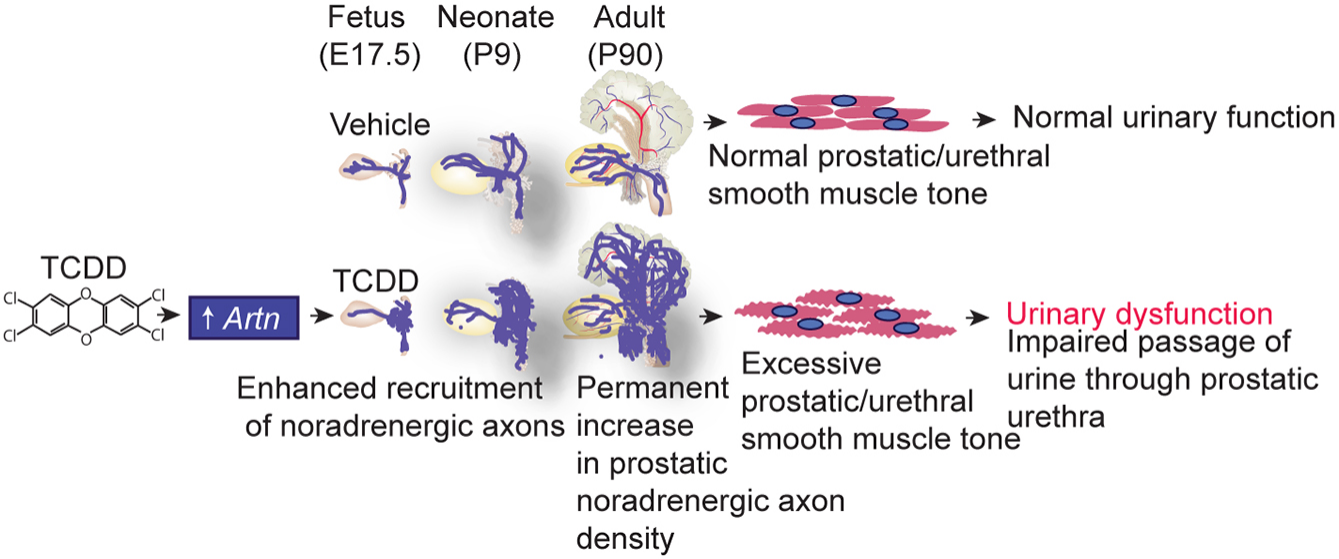
**Proposed mechanism underlying TCDD-induced urinary dysfunction in mice.** TCDD increases the abundance of the neurotrophic factor *Artn* in the developing prostate and enhances noradrenergic axon growth. These TCDD actions increase prostatic noradrenergic density in the fetus, neonate and adult, and lead to prostatic smooth muscle hyperactivity and urinary dysfunction in adulthood.

Our results support a model by which TCDD exposure during urogenital development in the fetus induces *Artn* to enhance noradrenergic axon recruitment to the developing prostate, thereby increasing the basis for a larger response to sympathetic nerve activation and driving a change in urinary function that persists into adulthood (Fig. 5). We propose this mechanism as a means by which the intrauterine environment can influence baseline urinary physiology and serve as a risk factor for LUTD in aging men.

## DISCUSSION

Chemical exposures during the fetal and neonatal periods influence risk of adult obesity, neurodevelopmental disorders and some forms of cancer (Levin et al., 1988; Grandjean and Landrigan, 2006; Carpenter and Bushkin-Bedient, 2013), but their impact on male urinary function during advancing age has largely been unexamined. Using perinatal exposure to a persistent environmental contaminant (TCDD) as a model for exposure to a range of common environmental contaminants that activate the AHR, we find that the intrauterine and early postnatal environment shapes prostate noradrenergic axon density, leading to lasting changes in prostate smooth muscle response and voiding function that persist into adulthood. It is unclear why prostate baseline tension, smooth muscle activity and responsiveness to α-adrenergic receptor blockers varies so considerably among human males with LUTD (Lee et al., 2017). We posit that maternal chemical exposures during fetal urogenital development may underlie this variability, creating a susceptible phenotype that sensitizes males to voiding dysfunction with advancing age. In support of this concept, TCDD exposure worsens urinary voiding dysfunction in mice challenged in adulthood with exogenous testosterone and estradiol implants (Ricke et al., 2016). Here, we show that TCDD exposure is, by itself, sufficient to change urinary voiding dynamics in the absence of exogenous hormone challenge and can predispose to voiding dysfunction, even in young adult mice. We describe increased noradrenergic axon density and prostate smooth muscle hyperactivity as a mechanism for abnormal voiding in adulthood and for hypersensitivity of TCDD-exposed mice.

Exposure of the fetus to TCDD during the period when noradrenergic axons are recruited into the prostate (Turco et al., 2019) increases abundance of *Artn*, a neurotrophic factor driving noradrenergic axon guidance and growth. ARTN mediates sympathetic axon growth along vasculature at the time of initial axon extension, induces sympathetic axon outgrowth *in vitro*, and attracts sympathetic axons *in vitro* and *in vivo* (Enomoto et al., 2001; Glebova and Ginty, 2005). Mice deficient in *Artn* exhibit abnormally short or misdirected proximal axons from sympathetic chain ganglia (Honma et al., 2002). More than 50% of sympathetic neurons generated in the embryo are killed by apoptosis during normal development (Oppenheim, 1991). One hypothesis for why excessive innervation is a widespread activity in the developing embryo is that neurons are initially overproduced and, once they innervate their target, compete for limited target-derived neurotrophic factors (Levi-Montalcini et al., 1954). Our data point to TCDD exposure increasing *Artn* expression, in turn leading to persistent hyperinnervation. We localized *Artn* within fetal mouse prostate and used independent methods and two separate mouse cohorts to show that TCDD increases *Artn* mRNA abundance in the fetal prostate. TCDD is a potent ligand of the AHR, a ligand-dependent transcription factor that mediates most, if not all, of TCDD's biological actions (Edamitsu et al., 2019). Promoting the idea that *Artn* induction is a more generalizable mechanism of AHR action, a recent study found that the activated AHR docks at the *Artn* gene locus, increases *Artn* expression and drives hyperinnervation of adult mouse skin (Hidaka et al., 2017).

A surprising observation in this study is the persistence of the prostate hyperinnervation phenotype induced by fetal TCDD exposure, beginning in the fetal period and continuing into adulthood. *Artn* is transiently upregulated at E17.5; however, we cannot rule out the possibility that TCDD-mediated increases in neurotrophic pathways at later time points also contribute to the persistent increase in prostatic axon density. Other neurotrophic factors such as neuropilin 1, neuropilin 2 and semaphorin 3a (SEMA3A) also control rat and mouse sympathetic axon growth, guidance and remodeling (Nangle and Keast, 2011; Maden et al., 2012; Richeri et al., 2020). TCDD acts through SEMA3A to disrupt the peripheral nervous system of the red seabream (Iida et al., 2013). It is also interesting that the TCDD-mediated transient increase in fetal prostatic *Artn* expression is associated with increased density of sympathetic, but not sensory, axons in the prostate. One possibility is that glial cell line-derived neurotrophic factor family receptor alpha 3 (GFRA3; a major receptor for ARTN) is more abundant in noradrenergic axons than in sensory and cholinergic axons of the developing prostate, a possibility that will be explored in future studies. Based on our findings, we predict that fetal exposure to AHR agonists would induce benign prostatic changes and LUTD without causing pelvic pain, although further investigation is required.

To further define the mechanism underpinning sympathetic hyperinnervation of the prostate, it would be of interest to investigate the potentially persistent effects of TCCD exposure on pelvic ganglia, as these are the primary source of sympathetic axons innervating the prostate and other pelvic organs. This approach could determine whether TCCD drives hyperinnervation by stimulating the postnatal maturation of pelvic ganglia (in which the number of neurons normally continues to increase in the first couple of weeks after birth). Alternatively, TCCD may also stimulate the growth of axon collaterals within the prostate. We previously observed that axon density in the mouse prostate continuously increases from the fetal period to adulthood (Turco et al., 2019). If chemical exposure causes excessive noradrenergic axons to grow within the developing prostate, and this aberrant growth persists postnatally and into adulthood, this may be a fundamental generalizable mechanism of urinary voiding dysfunction in aging men and influence the symptomatic response to inflammation and therapeutic response to α-adrenergic receptor blockers. It is important to note that several chemicals have been implicated in abnormal prostate development, including environmental, pharmaceutical and dietary estrogens (Saito et al., 2000; Timms et al., 2005), plasticizers (Moore et al., 2001) and bacterial toxins (Zhang et al., 2017), among others. Whether these other chemicals influence innervation of the prostate has not been determined. Investigation of the impact of other AHR agonists on prostate innervation would be of significant interest and relevance to LUTD.

## MATERIALS AND METHODS

### Mice

All mice were purchased from The Jackson Laboratory (Bar Harbor, ME, USA) and maintained on a C57BL/6J genetic background (stock #000664), *Polr2a^Tn(pb-CAG-GCaMP5g,-tdTomato)Tvrd^* (also known as *GCaMP5g*; stock #024477) and Tg(*Myh11cre*,-EGFP)2Mik (also known as *Myh11*^cre^; stock #007742). Mice were housed in Innovive^®^ HDPE plastic microisolator cages in a room maintained on a 12 h light and dark cycle, ambient temperature of 20.5±1°C, and relative humidity of 30-70%. Mice were fed a 5015 Diet (PMI Nutrition International, Brentwood, MO, USA) from conception through weaning (P21) and an 8604 Teklad Rodent Diet (Harlan Laboratories, Madison, WI, USA) thereafter. Food and water were available *ad libitum*, and cages contained corncob bedding. Mice were time-mated by pairing males and females overnight. The morning of definitive copulatory plug identification was considered E0. For all studies other than RNA-seq, mated females with a body weight increase of 4 g at 13 days post-breeding (indicating pregnancy) (Heyne et al., 2015) were given a single dose of TCDD [1 µg/kg, orally (po), 98% purity; Cambridge Isotope Laboratories, Andover, MA, USA] or corn oil (5 ml/kg, po; vehicle control) on E13.5. TCDD or vehicle was administered using a reusable 12-gauge, 1.5 inch animal feeding/oral gavage needle (VWR International, 20068-636). The TCDD dose used in this study matches that of a previous reference study (Moore et al., 2016). Based on the 11-day whole-body elimination half-life of TCDD in C57BL/6J mice, the TCDD dosing paradigm used in this study results in continuous TCDD exposure from the fetal period through weaning (Gasiewicz et al., 1983). All mice were euthanized by CO_2_ asphyxiation. UGS and prostate tissues were collected at E17.5, P9 and P90-P98, either snap frozen in liquid nitrogen or fixed overnight at 4°C in 4% paraformaldehyde dissolved in neutral buffered saline (Thermo Fisher Scientific) and dehydrated through a series of graded ethanol concentrations. Tissues were cleared in xylenes, embedded in paraffin wax and sectioned at 10 μm thickness on a microtome (Surgipath Medical Industries).

### Cystometry in anesthetized adult male mice

Cystometry was performed as previously described (Bjorling et al., 2015; Ritter et al., 2017; Ruetten et al., 2019). Mice were anesthetized with urethane [1.43 g/kg, subcutaneous (sc)], because isoflurane interferes with normal micturition, and rested for 30 min. Mice remained under urethane anesthesia for the duration of performance of cystometry. The deep plane of anesthesia was confirmed by repeatedly confirming the absence of response to toe pinch during cystometry. An incision was made in the ventral abdomen to expose the bladder. A purse string suture was placed in the bladder dome, and polyethylene cystometry tubing (PE-50; outer diameter, 0.58 mm; inner diameter, 0.28 mm) was inserted into the bladder through the center of the suture and secured. The abdominal wall and skin were closed, and the exterior tubing was sutured to the abdominal skin to prevent movement. Mice were allowed to recover on a heating pad for 1 h post procedure. The exposed tubing was attached to a three-way stopcock and connected to an infusion pump (Harvard Apparatus, Holliston, MA, USA) and pressure transducer (Memscap). Bladder pressure was simultaneously recorded using a PowerLab data collection system (ABInstruments, Colorado Springs, CO, USA). Saline (0.9%) was infused into the bladder at a rate of 1.5 ml/h. At least 1 h of voiding activity was recorded. Three to five consecutive voids, occurring after stabilization of micturition cycles, were used for analysis.

### Smooth muscle calcium transient imaging

*Myh11cre*/+;*GCaMP5g/*+ mice harbor a genetically encoded calcium sensor that fluoresces in response to increases in intracellular calcium concentration*. Myh11*^cre^/+;*GCaMP5g*/+ mice were euthanized at 14 weeks of age, and a tissue complex containing urethra and anterior, dorsal and ventral prostate was dissected and stored in ice-cold HEPES buffer (134 mM NaCl, 10 mM HEPES, 6 mM KCl, 7 mM glucose, 1 mM MgCl_2_, pH 7). The anterior prostate was dissected and analyzed separately, whereas the dorsal prostate was analyzed attached to the urethra due to its small size. The tissue was positioned in a heated (37°C) chamber for imaging (Warner Instruments, RC-49MFSH). A pressurized perfusion system (Warner Instruments, 641721) fitted with mini-valve controllers (Warner Instruments, VCS-8-MINI-LT) was used to continuously perfuse tissues with HEPES solution (134 mM NaCl, 10 mM HEPES, 6 mM KCl, 7 mM glucose, 1 mM MgCl_2_, 2 mM CaCl_2,_ pH 7; 1 ml/min), and maintained at 37°C with an inline heater (Warner Instruments, SF-28). The tissue was illuminated with an LED light (SOLA-SM2 365 LED Light Engine) and visualized with an MZ16 stereo fluorescent dissecting microscope (Leica, Wetzlar, Germany) fitted with a GFP dichroic filter set (Chroma, Bellows Falls, VT, USA). Images were recorded with a Zyla 4.2 Plus sCMOS camera using Nikon Elements Software. A Grass stimulator (model S88, pulse stimulator) was used to deliver electrical impulses for 10 s at 60 V and 0.1 Hz (Lau et al., 1998). After a 5 min recovery period, tissues were stimulated again for 10 s at 60 V and 30 Hz (Lau et al., 1998). Videos were recorded at 10 frames/s and analyzed using SparkAn (in-house software designed by Dr Adrian Bonev, University of Vermont, Burlington, VT, USA). Investigators were blinded to treatment group by labeling videos with exclusively mouse number and date.

### Isometric prostate muscle tension analysis

*In vitro* organ bath studies were conducted as previously described (Tengowski et al., 1997; Keil et al., 2015). Excised dorsal prostates and bladder were weighed, and 5-0 sutures were applied to secure the dorsal prostate and subdissected bladder strips at the base to the specimen arm and at the tip of the tissue to the force displacement transducer (Grass FT-03). Tension was recorded using a PowerLab data collection system (ABInstruments, Colorado Springs, CO, USA). The prostate and bladder were suspended in a 37°C water-jacketed tissue chamber filled with Krebs solution (133 mM NaCl, 16 mM NaHCO_3_, 5 mM KCl, 1 mM MgCl_2_, 1.4 mM NaH_2_PO_4_, 2.5 mM CaCl_2_ 2H_2_O, 7.8 mM D-glucose, pH 7.2) and aerated with 95% O_2_-5% CO_2_. Tissues were maintained at 0.7 g of tension for 60 min before experimentation, with Krebs solution changed every 15 min. Tension was recorded using AxoScope Application of PCLAMP software (Molecular Devices, Sunnyvale, CA, USA). Tissues were stimulated for 5 s at 0.1, 1, 3, 10, 30 and 60 Hz at 30 V, with 3 min between each stimulation. Tissue baths were flushed with Krebs solution, and tissues were allowed to return to baseline (0.7 g) tension for 30 min, after which a concentration response curve to the α-adrenergic agonist phenylephrine (0.0001, 0.01, 1, 100 and 200 µM) was performed. After washout and return to baseline (0.7 g) tension for 30 min, tissues were incubated with guanethidine for 15 min (White et al., 2010), before being stimulated as described above at 0.1, 1, 3, 10 and 30 Hz. After a final washout and return to baseline for 30 min, tissues were maximally contracted with 60 mM KCl. Tissue responses were normalized to maximum response to KCl and expressed as a percentage thereof. Investigators were blinded to treatment group by labeling videos with exclusively mouse number and date.

### Immunohistochemistry

Paraffin-embedded tissue sections (10 µm) were deparaffinized with xylene and rehydrated in a series of graded concentrations of ethanol. Immunofluorescence staining was conducted as described previously (Abler et al., 2011; Turco et al., 2019) using the antibodies listed in Table S1. Tissue sections were imaged using an SP8 confocal microscope (Leica) fitted with a 20× oil immersion objective (HC PL Apo CS2 NA=0.75; Leica). Ten *z*-stack images were captured at 1024×1024 resolution and a *z*-interval of 1 µm using LASX 8 software (Leica). The detector gain ranged between 10 and 30 using a Hy-D detector. All fluorescent images were stained using Alexa Fluor 488, Rhodamine Red and Alexa Fluor 647 secondary antibodies (Table S1). These fluorophores were excited using the 488 nm, 561 nm and 633 nm lasers, respectively, and images were collected sequentially. Whole-image RGB color intensity was adjusted using Adobe Photoshop only after quantification (version 20.0).

### *In situ* hybridization

RNA *in situ* hybridization was performed on 5 µm paraffin sections of mouse tissue using an RNAScope 2.5 HD Assay kit (Advanced Cell Diagnostics, 322360) following the manufacturer’s instructions. Optimized conditions for target retrieval were determined as a 15 min incubation in the target retrieval buffer and a 30 min treatment with proteinase K reagent. The probe against mouse *Artn* (800811) was obtained from the Advanced Cell Diagnostics probe catalog. Images were obtained at 20× on a Zeiss Axio Scan Z1 microscope and processed using Zen Blue software.

### Morphometric analysis

TH^+^, TUBB3^+^, VAChT^+^ and CGRP^+^ prostatic axons were quantified in E17.5, P9 and P90 prostate tissue sections, as previously described (Turco et al., 2019). For the embryonic stage, axons were quantified in the 10 µm stromal band extending from the basal surface of CDH1-stained UGS using ImageJ. For postnatal stages, axons were quantified in the 10 µm stromal band extending from the basal surface of CDH1-stained prostatic ductal epithelium (this region encompasses the majority of periductal smooth muscle). Immunostained axon pixels were quantified using the thresholding feature (MaxEntropy thresholding) of ImageJ (version 1.52e11) (Kapur et al., 1985). Image pixel density was scaled to 2.23 pixels/µm. For each age and sampled region, three non-adjacent, near-midsagittal, technical replicate tissue sections (10 µm) were averaged per mouse, and four to ten biological replicate mice were evaluated per axon subtype. Mice of each age were derived from at least three separate litters. Investigators were blinded to treatment group by labeling videos with exclusively mouse number and date.

### RNA sequencing of fetal prostate epithelium

Pregnant C57BL/6J mice were treated po with either 5 ml/kg corn oil (control) or 5 µg/kg TCDD on E13.5 following previously established protocols (Lin et al., 2003). Dams were euthanized at E16.75 via CO_2_ asphyxiation, and UGSs were dissected from fetuses, as previously described (Moore et al., 2016). Each UGS was immediately placed into 300 µl of 1% trypsin (Difco, 215240) in PBS and incubated on ice for 30 min. Collagenase (Sigma-Aldrich, C9891) was added to a final concentration of 1 mg/ml, followed by an additional 30-45 min incubation on ice. A dissecting microscope was used to mechanically separate UGS mesenchyme from UGS epithelium (UGE) and excise bladder and distal urethra, leaving only the portion of UGE from which the prostate derives. Each treatment group consisted of four biological replicates, and each replicate contained five or six individual UGEs that were pooled for RNA isolation. Total RNA was purified from each UGE using an RNeasy^®^ system (Qiagen) and analyzed using a Bioanalyzer 2100 and RNA 6000 PicoKit (Agilent Technologies). The samples were sequenced at the University of Wisconsin-Madison, Biotechnology Center, using an Illumina HiSeq^®^ 2500.

### RNA-seq analysis

Processing of RNA-seq data followed an updated pipeline for gene-level analysis (Anders et al., 2013; Garcia et al., 2018). Briefly, reads were evaluated by FastQC v0.11.9 (https://www.bioinformatics.babraham.ac.uk/projects/fastqc/) to detect major sequencing problems, and then trimmed for quality control with two rounds of Skewer v0.2.2 (Jiang et al., 2014) to remove ends of reads with low mean Phred quality score (Skewer round 1 options: x –AAAAAAAAAAAAAAAAAAAAAAAAAAAAAAAAAAAAAAAAAAAAAAAAAAAAAAAAAAAAAAAA -–mode pe -–max 100 -–cut3 -–format auto -–compress -–threads 9; Skewer round 2 options: x Mouse_adapters.fa– -–mode pe -–end-quality 30 -–mean-quality 30 -–min 30 -–format auto -–compress -–threads 9; Table S2). RNA-seq alignment and quantification proceeded with Bowtie2 v2.4.1 (Langmead et al., 2009; Langmead and Salzberg, 2012) being used to build HISAT2 v2.1.0 (Kim et al., 2015; Kim et al., 2019) genome index files from the Genome Reference Consortium Mouse Build 38 patch release 6 (GRCm38.p6) genome downloaded from Ensembl (release 100; https://ftp.ensembl.org/pub/release-100/fasta/mus_musculus/dna/Mus_musculus.GRCm38.dna.toplevel.fa.gz). To enhance the genome index files, the HISAT2 scripts hisat2_extract_splice_sites.py and hisat2_extract_exons.py were used to extract splice site and exon information, respectively, from the Ensembl 100 gene transfer format (GTF) file (https://ftp.ensembl.org/pub/release-100/gtf/mus_musculus/Mus_musculus.GRCm38.100.gtf.gz). The extracted genomic information was incorporated into HISAT2 index files, using the hisat2-build command. After building enhanced genome index files, trimmed reads were aligned with HISAT2 v2.1.0 and then piped through SAMtools v1.10 (Li et al., 2009) to convert aligned reads to binary alignment style (BAM) and then to sort BAM reads by position, using the following command: hisat2 –q -–phred33 -rna-strandness– RF -–no-mixed -–no-discordant -–new-summary -–threads 9 –x *GENOME_INDEX* −1 *PAIR1_FILE.fastq.gz* −2 *PAIR2_FILE.fastq.gz* | samtools view –u @9 | samtools– sort –n –O bam –o *OUTPUT_FILE_sorted_byName.bam* –@9. Gene counts were estimated using the htseq-count command from HTSeq v0.12.4 (Anders et al., 2015) with the GRCm38.p6 Ensembl 100 GTF annotation (options: -–format=bam -–order=name -–stranded=reverse -–type=exon -idattr=gene_id– -–additionalattr=gene_name– -–mode=intersection-nonempty).

Analysis of gene counts was conducted using R v4.0.2 (https://www.r-project.org/, accessed 12 February 2021) and Bioconductor v3.11 (Gentleman et al., 2004; Huber et al., 2015) packages in the RStudio v1.3.959 (https://www.rstudio.com/) integrated development environment with a customized script (Dataset 2) based on a maintained Bioconductor workflow package from the Gordon Smyth laboratory (https://www.bioconductor.org/packages/release/workflows/vignettes/RnaSeqGeneEdgeRQL/inst/doc/edgeRQL.html, last updated 13 June 2020; Chen et al., 2016). Counts from technical replicates split across sequencing lanes were averaged, and initial exploratory data analysis (i.e. gene variance heatmaps and PCA plots) revealed an outlier TCDD sample, which was removed. The Bioconductor package, edgeR v3.30.3, was used to normalize gene counts and determine differential expression (Robinson and Smyth, 2007, 2008; Robinson and Oshlack, 2010; Robinson et al., 2010; McCarthy et al., 2012; Lun et al., 2016). Briefly, genes were filtered using their filterByExpr function to exclude those with low counts in a minimum number of samples across libraries (Chen et al., 2016; Lun et al., 2016). Filtered genes were then normalized across samples using the trimmed mean of M values (TMM) method to minimize composition bias between libraries (Robinson and Oshlack, 2010). Differential expression of genes was determined using functions from edgeR, which uses the negative binominal generalized linear model extended by quasi-likelihood methods to fit the count data, the Cox–Reid profile-adjusted likelihood method to calculate dispersions, and empirical Bayes quasi-likelihood *F*-tests, while differential expression above a logarithmic fold change threshold of log_2_(1.5) between experimental and control samples was subsequently determined using a version of the *t*-tests relative to a threshold (TREAT) method (McCarthy and Smyth, 2009; McCarthy et al., 2012; Chen et al., 2014). The ‘robust=TRUE’ option was used to protect the empirical Bayes estimates against the possibility of outlier genes with wide ranging individual dispersions. Genes with a Benjamini–Hochberg FDR-adjusted *P*≤0.05 were considered significantly differentially expressed. The biomaRt v2.44.1 package was used to connect Ensembl gene identifier information to Ensembl BioMart annotation information (e.g. gene symbols, biotypes), and the volcano plot was made using ggplot2 and ggrepel (Durinck et al., 2005, 2009; Wickham, 2009; https://rdrr.io/cran/ggrepel/, accessed 12 February 2021). Heatmaps were made using the R packages dendextend v1.13.4 and ComplexHeatmap v2.4.2 (Durinck et al., 2005, 2009; Galili, 2015; Gu et al., 2016). Heatmap clustering was derived from TMM-normalized, variance-stabilized transformed gene values scaled by *z*-score (Love et al., 2015). Sequencing data and processing details have been deposited in the National Center for Biotechnology Information Gene Expression Omnibus (GEO; accession number GSE166395).

### RNA isolation and RT-PCR

Neonatal and fetal prostates were homogenized, as described previously (Tengowski et al., 1997). RNA was purified with an Illustra RNAspin minikit (GE Healthcare, Pittsburgh, PA, USA) and reverse transcribed with a SuperScript III First Strand Synthesis System (Invitrogen, Carlsbad, CA, USA). Real-time RT-PCR was performed in 10.5 µl reactions containing 1× SsoFast EvaGreen Supermix (Bio-Rad Laboratories, Hercules, CA, USA), 0.48 μM PCR primers and 3.75 µl cDNA, and amplified using a CFX96 PCR machine (Bio-Rad Laboratories). PCR primers are listed in Table S3, with *Ppia* used as an internal loading control. Relative mRNA abundance was determined by the deltaCt method, as described previously (Livak and Schmittgen, 2001).

### Statistics

The number of mice per group ranged from four to 11, and groups represented at least three independent litters. Power calculations were performed to determine the number of mice needed in each experiment. Statistical analysis was performed using GraphPad Prism 8 (version 8.2.1), and a difference between means was considered significant at *P*≤0.05. Differences among or between groups were identified using one-sided unpaired Student's *t*-test (intervoid interval, TH^+^, VaChT^+^, TUBB3^+^ axon density quantification, GCaMP analysis, tissue bath analysis, RT-PCR), one-way ANOVA (CGRP axon density quantification), repeated measures two-way ANOVA (frequency dose response, guanethidine quantification) and non-linear regression model (least squares, phenylephrine dose response). Bartlett's test was used to determine homogeneity of variance before using ANOVA to determine whether a parametric or nonparametric test could be used. Data that did not meet the criteria for homogeneity of variance or normality were transformed (log or square root). The Grubb's test identified extreme studentized deviates, and significant outliers were excluded from analysis. We concluded that these tissues sections were adjacent to an intramural ganglion, and they were removed from statistical analysis.

### Study approval

All procedures were approved by the University of Wisconsin Animal Care and Use Committee and conducted in accordance with the National Institutes of Health (NIH) Guide for the Care and Use of Laboratory Animals.

## Supplementary Material

Supplementary information
