# Supplementary material for: A mechanism linking perinatal 2,3,7,8 tetrachlorodibenzo-p-dioxin exposure to lower urinary tract dysfunction in adulthood
Source: Dis Model Mech. 2021 Jul 27;14(7):dmm049068. doi: 10.1242/dmm.049068 (PMC8326766; doi:10.1242/dmm.049068)
Supplement: Supplementary information [file dmm-14-049068-s1.pdf]

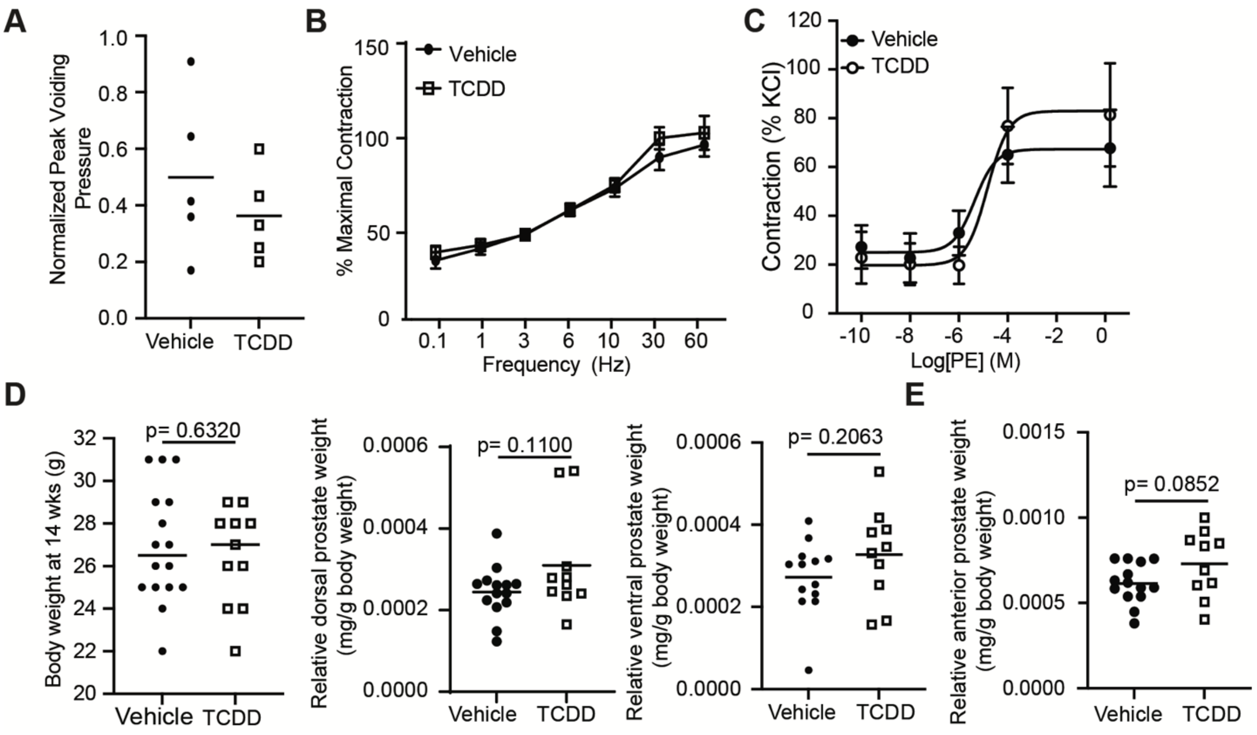

Fig. S1. TCDD exposure does not change body weight, prostate weight, response to alpha adrenergic agonist, or frequency dose response in bladder. (A) TCDD exposure does not change the peak voiding pressure during anesthetized cystometry. (B) TCDD exposure in the bladder does not significantly change the response to electrical field stimulation at any frequency (0.1-60 Hz) compared to vehicle. (C) TCDD exposure does not significantly shift the dose response to α-adrenergic agonist (phenylephrine), and the EC_{50} is the same for both groups (p = 0.8031, EC_{50} = 1.932). The baseline was set to 0.7g so tissues are considered baseline at 70%, shown by the dotted line. (D) *In utero* and lactational TCDD exposure does not change body weight (grams) at 14 weeks. (E) *In utero* and lactational TCDD exposure does not change the dorsal, ventral, or anterior prostate weight normalized to body weight. Students t-test was used to assess differences between groups in panel A, D, and E. Panel B an two-way ANOVA was used to analyze differences between groups at various frequencies. Non-linear regression analysis was used to analyze the phenylephrine dose response in panel C.

Disease Models & Mechanisms • Supplementary information

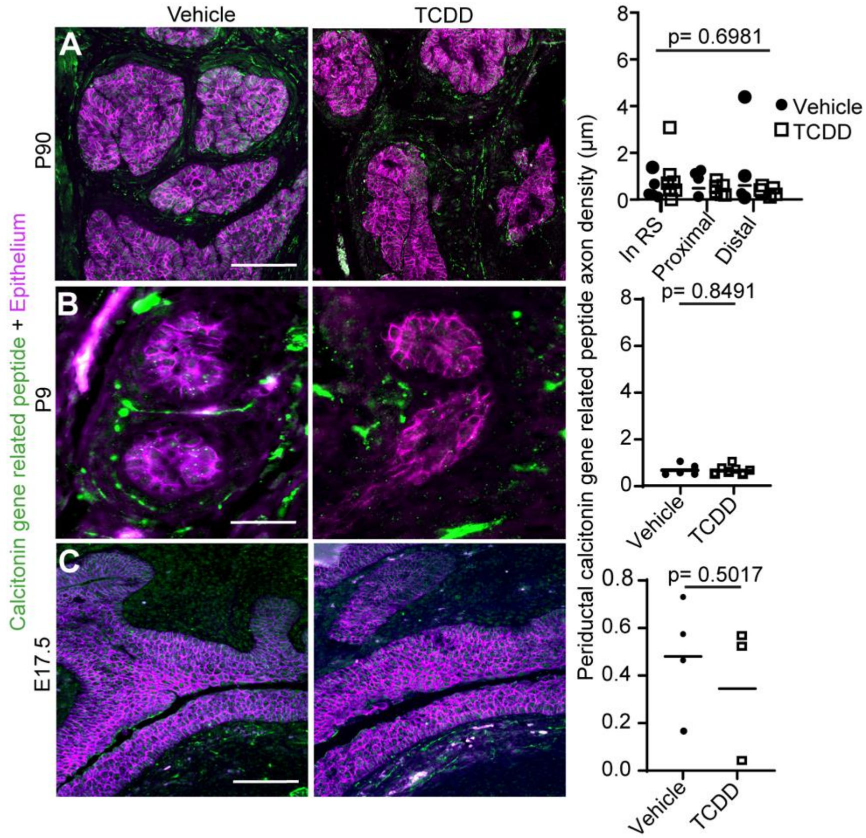

Fig. S2. TCDD does not change calcitonin gene-related peptide (CGRP+) axon density dorsal prostate. Pregnant C57BL/6J mice were dosed with TCDD (1 µg/kg maternal dose, po) or vehicle (5 mL/kg, po), and prostates were collected on E17.5, P9, and P90 (Panel A-C). P90 axon density was quantified in three distinct regions: inside the rhabdosphincter (In RS), proximal to the rhabdosphincter, and distal to the rhabdosphincter because sensory axon densities differ along the proximo-distal ductal axis. Prostate tissue sections were stained via immunohistochemistry to identify axons (green) and epithelium (magenta). The density of CGRP+ axons was quantified within a 10 µm band around the prostate ducts. The density of CGRP+ axons does not differ between vehicle and TCDD exposed mice. Results are from 4-7 mice per group belonging to at least three independent litters per group, and differences of $p \leq 0.05$ were considered significant. Students t-test was used to assess differences between groups. Scale bars represent 50 µm for P90 and P9 and 100 µm for E17.5.

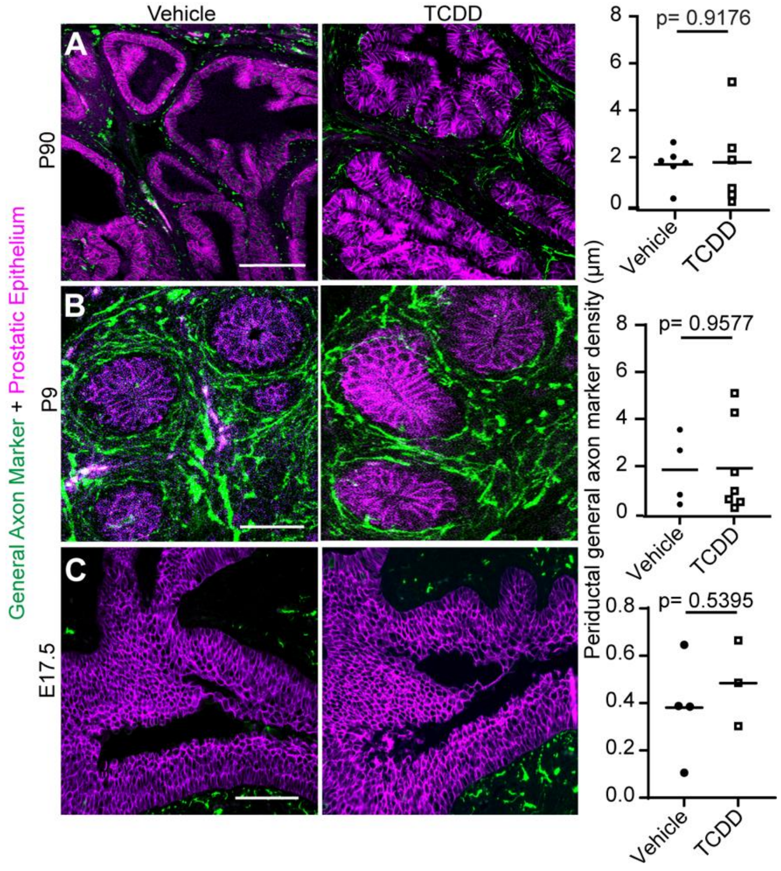

Fig. S3. TCDD does not change beta 3 tubulin (TUBB3+) axon densities in dorsal prostate. Pregnant C57BL/6J mice were dosed with TCDD (1 µg/kg maternal dose, po) or vehicle (5 mL/kg, po), and prostates were collected on postnatal day E17.5, P9, and P90. Prostate tissue sections were stained via immunohistochemistry to identify TUBB3 + axons (green) and epithelium (magenta). The density of TUBB3+ axons was quantified within a 10 µm band around the prostate ducts. The density of TUBB3+ axons does not differ between vehicle and TCDD exposed mice. Results are from 4-5 mice per group belonging to at least three independent litters per group, and differences of $p \leq 0.05$ were considered significant. Scale bar is 50 µm for P50 and P9 and 100 µm for E17.5. Student's t-test was used to assess differences between groups. Scale bars represent 50 µm for P90 and P9 and 100 µm for E17.5.

Disease Models & Mechanisms • Supplementary information

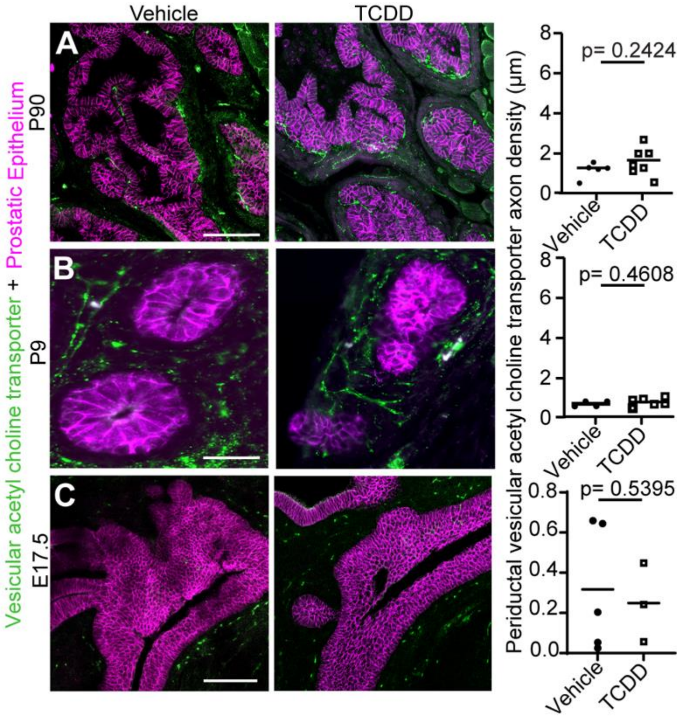

Fig. S4. TCDD does not change vesicular acetylcholine transporter (VAChT+) axon densities in dorsal prostate. Pregnant C57BL/6J mice were dosed with TCDD (1 µg/kg, po) or vehicle (5 mL/kg, po), and prostates were collected on E17.5, P9, and P90. Prostate tissue sections were stained via immunohistochemistry to identify axons (green) and epithelium (magenta). The density of VAChT+ axons does not differ between vehicle and TCDD exposed mice. Results are from 4-5 mice per group belonging to at least three independent litters per group, and differences of $p \leq 0.05$ were considered significant. Scale bar is 50 µm for P50 and P9 and 100 µm for E17.5. Student's t-test was used to assess differences between groups. Scale bars represent 50 µm for P90 and P9 and 100 µm for E17.5.

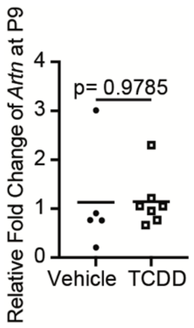

Fig. S5. Postnatal day 9 mouse prostatic *Arnt* mRNA abundance no longer differs between mice that were exposed to TCDD (1 µg/kg maternal dose, po) or vehicle (5 mL/kg, po) on E13.5. RT-PCR was performed on isolated P9 urogenital sinuses and normalized to *Ppia* abundance as an internal control. Results are from 6-7 mice deriving from at least three independent litters, and differences of $p \leq 0.05$ were considered significant. Students t-test was used to assess differences between groups.

Disease Models & Mechanisms • Supplementary information

Table S1. Antibodies

**Antibody**	**Vendor/Catalog Number**	**Antibody registry number (RRID)**	**Host Species**	**Dilution**	**Antibody Characterization**
Tyrosine hydroxylase (TH)	EMD Millipore/ AB152	AB_390204	Rabbit Polyclonal	1:300	(Van Kampen *et al.*, 2015)(Johnson *et al.*, 2015)(Bentea *et al.*, 2015)
Vesicular acetylcholine transporter (VAChT)	EMD Millipore/ ABN100	AB_2630394	Goat Polyclonal	1:250	(Turco *et al.*, 2019)
Calcitonin gene-related peptide (CGRP)	Sigma Aldrich/ C8198	AB_259091	Rabbit Polyclonal	1:100	(Turco *et al.*, 2019)
βIII tubulin (TUBB3)	Abcam/ab78078	AB_2256751	Rabbit Polyclonal	1:100	(Turco *et al.*, 2019)
E-Cadherin (CDH1)	Cell Signaling Technology/3195S	AB_2291471	Rabbit Monoclonal	1:200	(Vasilaki *et al.*, 2018)(Drubay *et al.*, 2018)(Lloyd-Lewis *et al.*, 2018)
E-Cadherin (CDH1)	BD Transduction Labs/610181	AB_39580	Mouse Monoclonal	1:100	(Wegner *et al.*, 2017)
Alexa Fluor 488-AffiniPure Anti-mouse IgG	Jackson ImmunoResearch 715-545-150	AB_2340846	Donkey	1:250	
Rhodamine-red-X AffiniPure Anti-Rabbit IgG	Jackson ImmunoResearch 711-295-152	AB_2340613	Donkey	1:250	
Alexa Fluor 647-AffiniPure Anti-Rabbit IgG	Jackson ImmunoResearch 711-605-152	AB_2492288	Donkey	1:250	
Alexa Fluor 488-AffiniPure Anti- Goat IgG	Jackson ImmunoResearch 705-545-003	AB_2340428	Donkey	1:250	

Table S2. FASTA sequences used in Mouse_Adapters.fa for Skewer adapter trimming

```
>Illumina Multiplexing Adapter 1
GATCGGAAGAGCACACGTCT
>Illumina_TruSeq_Adapter_Index_1
GATCGGAAGAGCACACGTCTGAACTCCAGTCACATCACG
>Illumina_TruSeq_Adapter_Index_2
GATCGGAAGAGCACACGTCTGAACTCCAGTCACCGATGT
>Illumina_TruSeq_Adapter_Index_3
GATCGGAAGAGCACACGTCTGAACTCCAGTCACTTAGGC
>Illumina_TruSeq_Adapter_Index_3a
ATCGGAAGAGCACACGTCTGAACTCCAGTCACTTAGGC
>Illumina_TruSeq_Adapter_Index_4
GATCGGAAGAGCACACGTCTGAACTCCAGTCACTGACCA
>Illumina_TruSeq_Adapter_Index_5
GATCGGAAGAGCACACGTCTGAACTCCAGTCACACAGTG
>Illumina_TruSeq_Adapter_Index_6
GATCGGAAGAGCACACGTCTGAACTCCAGTCACGCCAAT
>Illumina_TruSeq_Adapter_Index_7
GATCGGAAGAGCACACGTCTGAACTCCAGTCACCAGATC
>Illumina_TruSeq_Adapter_Index_8
GATCGGAAGAGCACACGTCTGAACTCCAGTCACACTTGA
>Illumina_read2_adapter_segment
AGATCGGAAGAGCGTCGTGTAGGGAAAGAGTGTAGATCTCGGTGGTCGCCG
>Illumina_read2_adapter_segment01
GATCGGAAGAGCGTCGTGTAGGGAAAGAGTGTAGATCTCGGTGGTCGCCG
>Illumina Multiplexing Index Read Sequencing Primer
GATCGGAAGAGCACACGTCTGAACTCCAGTCAC
>Illumina_read_adapter_segment_1
AGATCGGAAGAGCACACGTCTGAACTCCAGTCAC
>Illumina_read_adapter_segment_2
AGATCGGAAGAGCGTCGTGTAGGGAAAGAGTGTA
```

Table S3. RT-PCR primers

Target	NM Number	Left Primer	Right Primer
Artn	NM_011037.4	CTGGACCCAATGTCCCGCAG	AGTGTGTCCCCCTACCAGGC
Ppia	NM_008907.2	TCTCTCCGTAGATGGACCTG	ATCACGGCCGATGACGAGCC

Disease Models & Mechanisms • Supplementary information

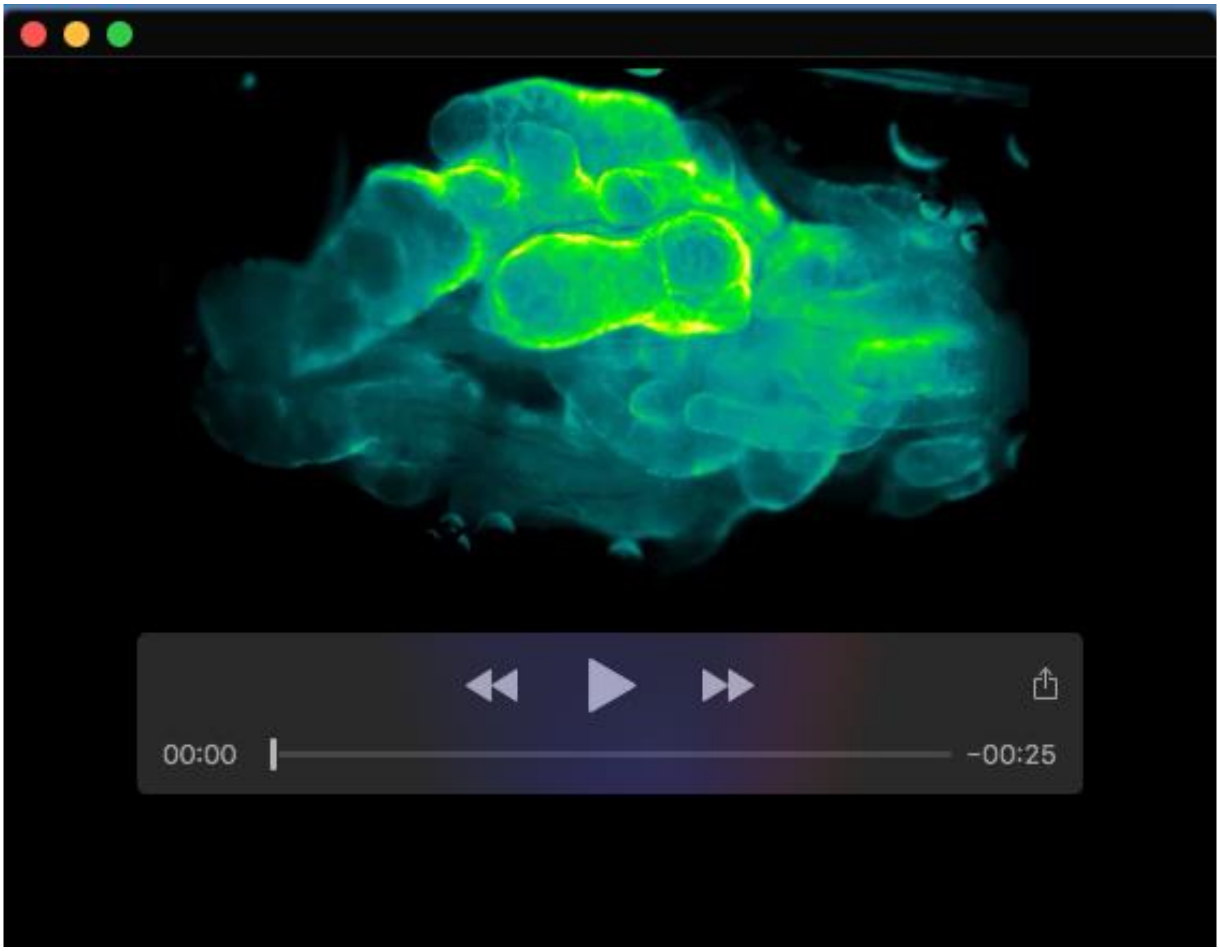

Movie 1. *V*ehicle-exposed prostate tissue stimulated with 0.1 Hz and 60 V for 10 seconds and imaged at 20X. Video is pseudocolored, with yellow showing maximal contraction and blue showing minimal.

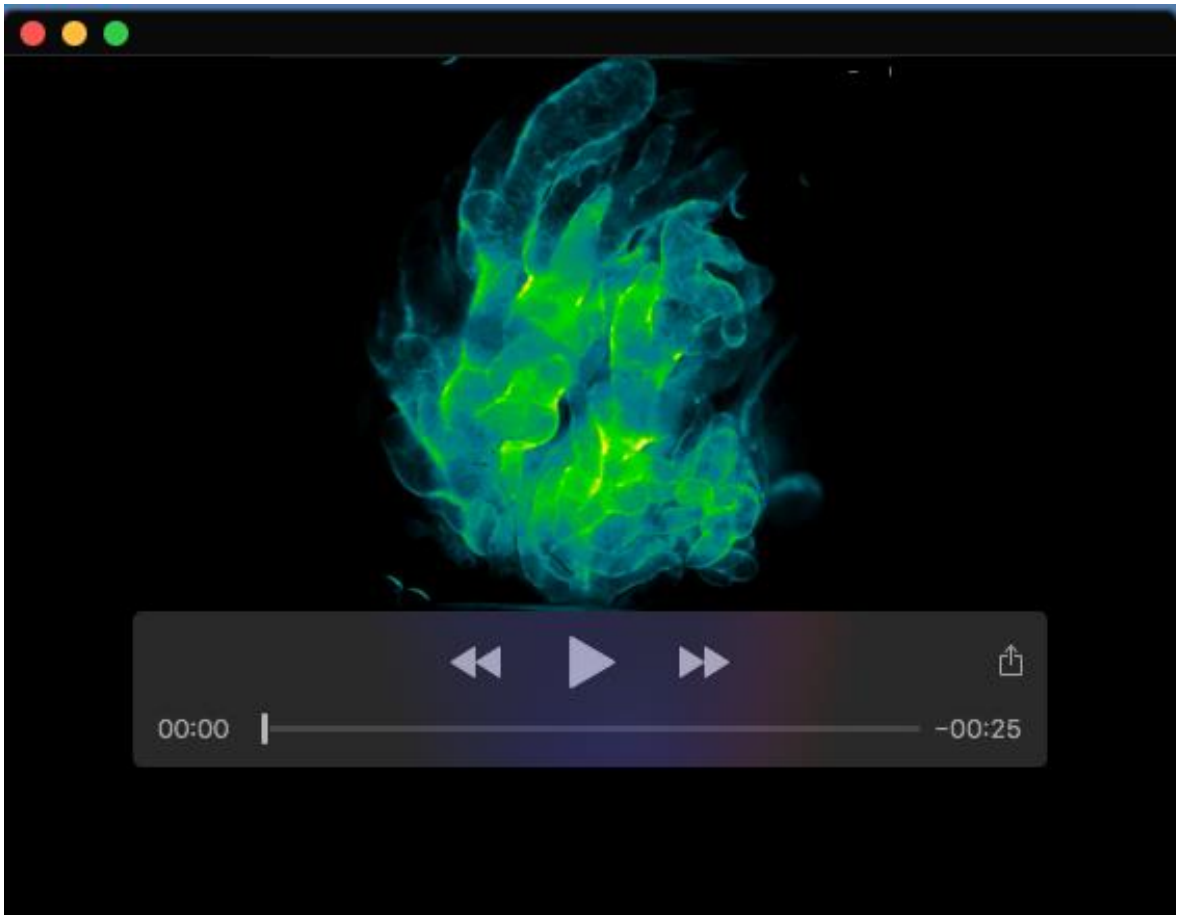

Movie 2. TCDD-exposed prostate tissue stimulated with 0.1 Hz and 60 V for 10 seconds and imaged at 20X. Video is pseudocolored, with yellow showing maximal contraction and blue showing minimal.

Dataset 1. Differential gene expression results as determined by edgeR.

Click here to download Dataset 1

Dataset 2. Differential gene expression R script.

Click here to download Dataset 2

Disease Models & Mechanisms • Supplementary information
